# Compartmentation and complexation of metals in hyperaccumulator plants

**DOI:** 10.3389/fpls.2013.00374

**Published:** 2013-09-20

**Authors:** Barbara Leitenmaier, Hendrik Küpper

**Affiliations:** Fachbereich Biologie, Universität KonstanzKonstanz, Germany

**Keywords:** hyperaccumulator/hypertolerance, excluder, metal sequestration, metal transport, metal storage cells, organic acids, phytochelatin

## Abstract

Hyperaccumulators are being intensely investigated. They are not only interesting in scientific context due to their “strange” behavior in terms of dealing with high concentrations of metals, but also because of their use in phytoremediation and phytomining, for which understanding the mechanisms of hyperaccumulation is crucial. Hyperaccumulators naturally use metal accumulation as a defense against herbivores and pathogens, and therefore deal with accumulated metals in very specific ways of complexation and compartmentation, different from non-hyperaccumulator plants and also non-hyperaccumulated metals. For example, in contrast to non-hyperaccumulators, in hyperaccumulators even the classical phytochelatin-inducing metal, cadmium, is predominantly not bound by such sulfur ligands, but only by weak oxygen ligands. This applies to all hyperaccumulated metals investigated so far, as well as hyperaccumulation of the metalloid arsenic. Stronger ligands, as they have been shown to complex metals in non-hyperaccumulators, are in hyperaccumulators used for transient binding during transport to the storage sites (e.g., nicotianamine) and possibly for export of Cu in Cd/Zn hyperaccumulators [metallothioneins (MTs)]. This confirmed that enhanced active metal transport, and not metal complexation, is the key mechanism of hyperaccumulation. Hyperaccumulators tolerate the high amount of accumulated heavy metals by sequestering them into vacuoles, usually in large storage cells of the epidermis. This is mediated by strongly elevated expression of specific transport proteins in various tissues from metal uptake in the shoots up to the storage sites in the leaf epidermis. However, this mechanism seems to be very metal specific. Non-hyperaccumulated metals in hyperaccumulators seem to be dealt with like in non-hyperaccumulator plants, i.e., detoxified by binding to strong ligands such as MTs.

## INTRODUCTION

Many heavy metals such as copper, manganese, nickel, and zinc are well-known as essential trace elements for all living organisms, mainly as active centers of enzymes. And surprisingly even cadmium, which never has been thought to be essential for any organism, has been found to be a micronutrient for the marine alga *Thalassiosira weissflogii*, where it builds up the active center of a carbonic anhydrase (Lane and Morel, 2000). For land plants no essential role for cadmium is known to date, although it has been shown to be beneficial for Cd hyperaccumulators as a defense against pathogens and herbivores (Jiang et al., 2005), and in the southern French populations of the Cd/Zn hyperaccumulator *Noccaea* (=*Thlaspi*) *caerulescens* (Brassicaceae) even a growth reduction in absence of Cd was observed without influence of pathogens or herbivores (Liu et al., 2008).

In many places worldwide, heavy metals can occur in very high concentrations that are detrimental or even lethal to most plant species. These high concentration are sometimes due to natural factors, leading to natural metallophyte communities, for example in special regions in Africa like the “copper belt” in the Republic of Congo where copper ore naturally comes to the surface (e.g., Duvigneaud, 1958; Malaisse et al., 1999). In many other cases, however, they are caused by human activities (e.g., Romic and Romic, 2003). As prominent examples, they are known to be results of mining (and smelters), where cadmium is often found together with zinc (Buchauer, 1973; Van Geen et al., 1997; Liu et al., 2005), and also high amounts of copper in the soil are found in mining areas (Ke et al., 2007). The same applies for manganese in parts of Southern China (Li et al., 2007). But also application of sewage sludge (McBride et al., 1997) and dust from car tires (Cd is a softener for rubber) along roadsides contributed to Cd pollution (Fergusson et al., 1980). Metal pollution is also found in rivers all over Europe (Audry et al., 2004), especially in regions rich in vineyards, as copper is still used as a classical reagent against fungal attacks toward vine plants (Küpper et al., 1996; Ribolzi et al., 2002; He et al., 2009; Mackie et al., 2012; Ruyters et al., 2013).

**FIGURE 1:**
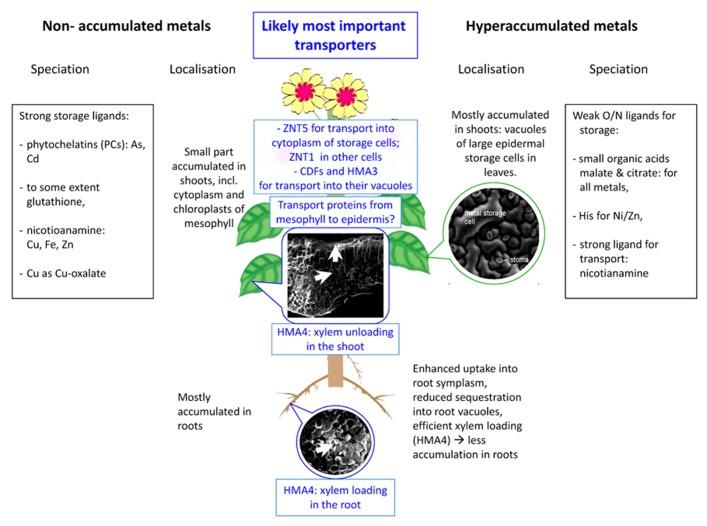
**Scheme of selected important aspects of metal sequestration and speciation in hyperaccumulated compared to non-hyperaccumulated metals – the latter includes non-accumulated metals in hyperaccumulators (e. g., copper in the Cd/Zn hyperaccumulator *Noccaeacaerulescens*.** The SEM micrographs show typical tissue morphology in hyperaccumulators and were taken during studies of HK. Epidermis with stomata: *Noccaea goesingense*; leaf cross-section:* Alyssum bertolonii*; root cross-section: *Arabidopsis halleri*.

Based on their ability to cope with metals in the medium they grow on/in, plants can be divided into three types:

(1) Indicator plants are usually sensitive to heavy metals. They can be used as an indicator for metal in the soil because the internal heavy metal concentration is a linear function of the bioavailable heavy metal concentration in the soil (or water for aqueous plants/nutrient solution for hydroponics).
(2) Excluders are a type of plants being able to tolerate heavy metals in the soil up to a threshold concentration by preventing the accumulation of metal in the cells. This exclusion is achieved either by blocking the uptake in the roots (see, e.g., Lux et al., 2011 for a detailed review) or by active (energy dependent) efflux pumps (Baker, 1981; van Hoof et al., 2001). Most metal (hyper)tolerant plants belong to this group (Baker, 1981).
(3) Most important for this topic are plants that can not only tolerate high concentrations of specific elements, but take them up actively and accumulate them to several percent of the dry mass of their above-ground parts. Originally described by Sachs (1865), since 1977 those plants are called hyperaccumulators (Brooks et al., 1977). Many studies on many metals, plant and pathogen species have shown that the hyperaccumulation phenotype naturally serves as a defense against pathogens and herbivores (Boyd and Martens, 1994; Martens and Boyd, 1994; Boyd et al., 2002; Hanson et al., 2003; Jhee et al., 2005 and many further studies since then, discussed in detail, e.g., in the reviews by Küpper and Kroneck, 2005, 2007). In this context, it should be mentioned that hyperaccumulation and hypertolerance are, according to studies on several hyperaccumulator species, genetically independent characters (Macnair et al., 1999; Bert et al., 2003). However, this “independence” is limited because a hyperaccumulator would poison itself if the accumulated metal would not be tolerated via specific mechanisms. More recent works also genetically question this independence, showing partially overlapping quantitative trait loci for accumulation and tolerance (Frérot et al., 2010; Willems et al., 2010). Hyperaccumulators have been found for many heavy elements and within many groups of plants and algae, including at least the following: Al, As, Cd, Cu, I, Mn, Ni, Se, Zn. More hyperaccumulators and hyperaccumulated elements are likely to be found as screening for this phenotype is ongoing and there is still a lot of debate about the definition of hyperaccumulation thresholds. However, nickel hyperaccumulators are by far the most numerous group (several hundred species are known) and biotechnology-oriented research focused on those metals where phytomining and/or phytoremediation are attractives uses, i.e., As, Cd, Cu, Ni, and Zn (see recent reviews, e.g., by Whiting et al., 2004; Chaney et al., 2005, 2007; Küpper and Kroneck, 2005, 2007). In this review, also we put most emphasis on the latter group of metals, but mention other hyperaccumulated elements as well when their complexation and/or compartmentation is different from this "core group" of hyperaccumulated elements. Furthermore, it should be noted that the mechanisms leading to accumulation of metals in hyperaccumulators are specific for one element or at maximum a few elements (see **Figure 1** for a short comparison between accumulated and non-accumulated metals). For example, while *N. caerulescens* accumulates up to several percent of its dry mass of both zinc and (only in case of the southern French populations) >2% of cadmium as well, its accumulation of copper is not elevated compared to non-accumulator species (Mijovilovich et al., 2009), the same applies to As, and it accumulates even less Mn than excluder species (Martïnez-Alcalá et al., 2013). Thus it is important to mention that the hyperaccumulation ability always depends strongly on the population and the metal – it is not possible to say that a specific species as such is a hyperaccumulator.

Depending on the type of plant, a number of strategies to resist the toxicity of heavy metals are known (for a quick summary, see **Figure 1**). In general, metal tolerance mechanisms include sequestration in specific cell types and intracellular compartments where metals can do the least harm, changing the speciation to less toxic forms by redox mechanisms, precipitation, or binding of metals to ligands, as well as export of metals out of the cells and plants. Since in principle this has been reviewed earlier (Prasad and Hagemeyer, 1999; Cobbett and Goldsbrough, 2002; Küpper and Kroneck, 2005; Verbruggen et al., 2009; Krämer, 2010 for general reviews; for cadmium accumulation Küpper and Leitenmaier, 2013), the current review will focus on recent new developments in this field that are relevant for a range of different metals.

As the most important mechanisms of metal tolerance in hyperaccumulating plants, compartmentation in specific storage cells as well as various ligands for detoxification and storage of metals will be discussed in the following. Furthermore, differences in metal compartmentation/complexation between hyperaccumulation and non-hyperaccumulating metal metabolism will be highlighted (for an overview, see also **Figure 1**).

## COMPLEXATION

In non-hypertolerant indicator plants, binding of metals by strong ligands is the main detoxification strategy. The best known type of ligands for this purpose are thiols, including glutathione, phytochelatins (PCs), and metallothioneins (MTs). In addition, some non-thiol ligands are involved, and the non-thiol ligands have a decisive role in hyperaccumulators. Already in 1996 histidine had been shown to be a main ligand of Ni in the Ni hyperaccumulator *Alyssum lesbiacum* (Krämer et al., 1996). In more recent times, the non-proteogenic amino acid nicotianamine has been shown to be involved in metal accumulation. Nicotianamine, glutathione, and MTs also have other functions for non-hyperaccumulated metals, such as transient binding during transport to the storage sites, storage and metal re-mobilization at times when metals are needed for active centers of enzymes, etc. PCs, in contrast, seem to be synthesized specifically for metal(loid) detoxification, particularly in the case of As and Cd (reviewed by Cobbett and Goldsbrough, 2002).

### CYSTEINE

Cysteine is well-known to be the most important metal-binding amino acid residue in proteins. Furthermore, as discussed below, it is a building block of PCs, the main As and Cd detoxifying compounds in non-hyperaccumulator plants. As a free amino acid, however, it has not been identified to be involved in binding metals for detoxification.

### HISTIDINE

Histidine is, besides the thiol ligand cysteine, the second most important amino acid residue forming metal-binding sites in metalloproteins, and both residues often are combined in the same site. Less known, however, is the role of the free amino acid histidine as a metal ligand. It was first shown to bind a major proportion of Ni in the Ni hyperaccumulator *Alyssum lesbiacum* (Krämer et al., 1996), but later it was found to be involved in binding zinc in hyperaccumulators as well (Salt et al., 1999; Küpper et al., 2004). In *Alyssum lesbiacum*, histidine was suggested to enhance loading of Ni into the xylem by a co-release of Ni and His into the xylem (Kerkeb and Krämer, 2003). In this context it is important to say that free histidine as a simple monodentate ligand is not binding metals very strongly, in contrast to the multi-histidine residue metal-binding sites in proteins. In this way, free histidine is important as a ligand most likely only in compartments where stronger ligands are scarce, e.g., the vacuole, xylem, or transport vesicles inside the cytoplasm, although low pH might limit complexation of metals by histidine in the vacuole and xylem. In the phloem, also stronger metal ligands (MTs, see below) were found, but histidine might bind metals there if the higher affinity sites are occupied. In the cytoplasm itself, in contrast, histidine most likely is an important ligand only as an amino acid residue of metal-binding proteins unless these high-affinity sites are already saturated with an excess of metal and all other detoxification ligands would be occupied (i.e., conditions of severe toxicity). Because of this, an alternative explanation seems more likely for the results of Richau et al. (2009). These authors demonstrated that addition of histidine to roots of *N. caerulescens* enhances loading of the xylem with Ni, but can block loading of Ni into isolated vacuoles. From the latter observation they concluded that Ni would bind to free His in the cytoplasm. In view of the facts discussed above, it seems more likely that His did not bind Ni directly in the cytoplasm, but in transport vesicles. Metal-rich vesicles have been observed in the cytoplasm of cells of hyperaccumulators (Neumann and De Figuero, 2002; Leitenmaier and Küpper, 2011), but so far it was not possible to analyze metal speciation in such small compartments.

### NICOTIANAMINE

As a polydentate ligand with three carboxyl groups and three nitrogens, this molecule is a rather strong ligand with pKs of 12.1 for Fe^2^^+^, 14.7 for Zn^2^^+^, 16.1 for Ni^2^^+^, and 18.6 for Cu^2^^+^ (Benevš, 1983), structurally very similar to the iron phytosiderophore mugeinic acid. However, for Zn and Fe these binding constants are still lower than those of typical metal-centers in metalloproteins. Nicotianamine provides six alternating carboxylate and amine functions; their relative positions favor the formation of six-coordinate metal complexes (Callahan et al., 2006). Its main role is still a matter of debate, it has been associated originally with iron metabolism (it binds both iron(II) and iron(III); Scholz et al., 1992; von Wiren et al., 1999). Soon after, it was shown to be important for copper metabolism (Pich et al., 1994; Pich and Scholz, 1996; Liao et al., 2000; Irtelli et al., 2009), and it was found to bind a significant proportion of total copper at physiologically relevant toxic concentrations in *N. caerulescens* (that does not hyperaccumulate copper; Mijovilovich et al., 2009). Also binding of zinc to nicotianamine was shown *in vivo* (Trampczynska et al., 2010). The latter, combined with elevated expression of NA synthase in the Cd/Zn model hyperaccumulators *Arabidopsis halleri* and *N. caerulescens* (Becher et al., 2004; Weber et al., 2004; van de Mortel et al., 2006, 2008) led to the question whether it may play a role in long-distance zinc transport in hyperaccumulators and therefore be an essential part of the hyperaccumulator phenotype. This hypothesis was farther supported by the finding that Ni in the xylem sap of *N. caerulescens* is partially present as stable Ni–NA complex (Mari et al., 2006). Recently, knocking down nicotianamine synthase by RNAi technology in *Arabidopsis halleri* has revealed that in such plants Zn and Cd accumulation is severely diminished, indicating that NA is indeed important for hyperaccumulation of these metals (Deinlein et al., 2012).

Taking the information above together, when present in low concentrations nicotianamine likely is important as a ligand mostly in compartments with low abundance of stronger ligands (e.g., the vacuole, xylem, and phloem). At the very high nicotianamine levels in hyperaccumulators, however, it might also take part in binding of these metals in the cytoplasm, although direct evidence for that [e.g., extended X-ray absorption fine structure (EXAFS) spectroscopy with subcellular resolution on intact frozen-hydrated tissues] is still missing because it is currently not yet possible due to technical reasons.

### OTHER NON-THIOL LIGANDS/DETOXIFYING ANIONS

While histidine and nicotianamine are becoming more known as relevant metal ligands in plants, also other non-thiol ligands have been shown to make a major contribution in particular cases, and it is possible that their actual importance is grossly underestimated. One such example are anthocyanins, which have been shown to be associated with molybdenum accumulation (Hale et al., 2001). Another such case is oxalate, which was first shown to bind copper in copper (Cu)-tolerant lichens and fungi (Chisholm et al., 1987; Fomina et al., 2005; Purvis et al., 2008) and also has been reported [by X-ray absorption spectroscopy (XAS) studies] to be the major ligand for manganese in *Phytolacca acinosa* Roxb. and *Phytolacca americana* (Phytolaccaceae; Dou et al., 2009; Xu et al., 2009). Furthermore, oxalate turned out to contribute a major part to copper binding in the copper sensitive Cd/Zn hyperaccumulator *N. caerulescens*, particularly in the more Cu-tolerant individuals of the population (Mijovilovich et al., 2009). While binding to oxalate will really detoxify copper and manganese, since copper and manganese oxalates are hardly soluble and a rather stable complex, binding to smaller organic acids like malic or citric acid will not significantly diminish bioavailability of toxic metals. For this reason, at first glance it is surprising that in various hyperaccumulator plants most of the hyperaccumulated metal(loid)s are associated with such organic acids (As: Wang et al., 2002, Cd: Küpper et al., 2004; Cu: Küpper et al., 2009; Mn, in various species outside the family of *Phytolaccaceae*: Fernando et al., 2010; Ni: Sagner et al., 1998; Zn: Salt et al., 1999). It is logical, however, in context with the compartmentation of metals in vacuoles of such plants, see next chapter of this review for details. In these compartments, in all plants high levels of malate and citrate are stored, so that storage of the metal(loid)s in the same compartment will inevitably lead to a weak association of both.

Organic acid ligands may also play a role in enhanced uptake of metals into the roots of hyperaccumulators. A recent study (Li et al., 2012) has shown that dissolved organic matter (DOM) in the rhizosphere of a hyperaccumulator ecotype of *Sedum alfredii* contained more organic acids and mobilized zinc better than DOM from a non-accumulator ecotype of *S. alfredii*.

### METALLOTHIONEINS

Metallothioneins are the best known metal detoxifying ligands, because they have been known from animal physiology for a very long time (reviewed by Cobbett and Goldsbrough, 2002). Soon, they were found in plants as well, although in less variants than in animals (for a review, see Freisinger, 2011). Their role in plants, however, seems to be significantly different from animals. In plants (non-hyperaccumulator plants as well as hyperaccumulators), their main role seems to be metal homeostasis under normal, non-toxic physiological conditions. For example, upregulation of MTs has been shown to occur during senescence of plant tissues for re-mobilization of copper (original research: Buchanan-Wollaston, 1994; Guo et al., 2003; Heise et al., 2007), and MT expression has been shown in the phloem (Vilaine et al., 2003; Barnes et al., 2004). Furthermore, Zn-MTs seem to be involved in physiological Zn deposition in developing seeds. Here, the best studied case is the MT Ec1 from wheat (Kawashima et al., 1992; Peroza and Freisinger, 2007; Loebus et al., 2011). In terms of detoxification, they seem not to be very important for Cd, but do play a role for Cu (reviewed by Cobbett and Goldsbrough, 2002). The study of Garcia-Hernandez et al. (1998) suggested that MT1 is important for scavenging copper mainly in leaf veins; as copper-induced MT1 expression was observed mainly in leaf veins and to a lesser extent in mesophyll cells, while hardly any induction of MT1 in response to copper stress was observed in roots.

In hyperaccumulator plants, MTs are not important for storage of the hyperaccumulated metals, as these are generally not bound by thiol ligands (see other Non-Thiol Ligands/Detoxifying Anions). Nevertheless, they were found to be overexpressed in the Cd/Zn hyperaccumulator *N. caerulescens* compared to related non-accumulators (Papoyan and Kochian, 2004). In our opinion, the most likely reason for this seemingly contradictory facts is detoxification of copper by MTs in hyperaccumulators of other metals (e.g., Cd, Zn, Ni) that are sensitive toward copper (Walker and Bernal, 2004; Mijovilovich et al., 2009). This is likely important for Cd/Zn hyperaccumulation, because due to the Irving–Williams series of binding strength of metals to ligands, also Cu will inevitably be transported by the in Cd/Zn hyperaccumulators highly overexpressed Cd/Zn transport proteins (Pence et al., 2000; Frausto da Silva and Williams, 2001). This copper transport was experimentally shown for the Cd/Zn-ATPase HMA4 (Leitenmaier et al., 2011). This unintended but unavoidable transport of Cu would lead to copper accumulation and copper toxicity in Cd/Zn hyperaccumulators if those species would not have mechanisms to eliminate excess copper. Thus we hypothesize that Cu binding by MTs is a step in this Cu detoxification and finally elimination. MTs were shown to bind Cu in these plants under relevant conditions in the Cd/Zn hyperaccumulator *N. caerulescens* (Mijovilovich et al., 2009), and for chemical reasons likely in other Cd/Zn hyperaccumulators. In that study, in the EXAFS analyses clearly a strong contribution of sulfur ligands to binding of copper was found in most of the samples. Further, copper(I) MTs are known to form very rigid copper–sulfur clusters (Calderone et al., 2005) that stabilize copper(I) in aqueous solution (Byrd et al., 1988), yielding characteristic multiple scattering signals in EXAFS spectra as they were detected in the study of Mijovilovich et al. (2009). The role of MTs in copper detoxification in hyperaccumulators is further supported by studies on MT3 in *N. caerulescens*, which showed enhanced copper binding capabilities (Roosens et al., 2004; Fernandez et al., 2012). The pathway of copper efflux in Cd/Zn hyperaccumulators after the binding to MTs, which must exist as they do not accumulate copper (Mijovilovich et al., 2009), is not yet known.

### PHYTOCHELATINS

Phytochelatins were discovered as heavy metal detoxifying ligands almost 30 years ago (Grill et al., 1985, 1989). In contrast to MTs, PCs seem not to be involved in normal metal metabolism under non-toxic conditions; neither a function in delivery of metals to metalloproteins nor in the storage of essential metals has been convincingly shown. Biochemically inhibiting (Schat et al., 2002) or genetically inducing PC synthase deficiency (cad1 mutant: Howden et al., 1995) makes non-hypertolerant plants hypersensitive to metal(loid) toxicity. Furthermore, many studies in non-hypertolerant plants have shown the induction of PC synthase by very low concentrations of metal(loid)s, in particular Cd and As (e.g., Hartley-Whitaker et al., 2001, and very recently Andresen et al., 2013), as they frequently occur in the environment. Thus, in our opinion on the basis of the evidence available, it is not enigmatic why PC synthase is present in all plants. Additionally, it is important to note that lack of PC synthase expression (the cad1 mutant) specifically leads to Cd hypersensitivity, without disturbance of growth at really low Cd (Howden et al., 1995). At “0” Cd in wildtype, these and other authors found about 3% of the PC level present at 30 μM Cd, which might be interpreted as an indication that PCs are needed even without Cd stress. However, all chemicals, labware, and water contain trace amounts of Cd, easily leading to nanomolar Cd concentrations in nutrient solution that were recently found to induce an increased PC synthesis (Andresen et al., 2013). In the latter study, PC levels dropped to almost undetectable levels if better than ACS grade chemicals (e.g., Suprapur/TraceSelect), ultraclean water and acid washed labware were used to push minimum Cd levels below 1 nM. Thus, the majority of data available so far strongly suggests that PC synthase is required only for metal(loid) detoxification. It has been reported that PC synthase contributes to degradation of glutathione conjugates (Blum et al., 2007; Wünschmann et al., 2010; Rigouin et al., 2013), but since PC synthase deficient mutants have no diminished growth when growing without As/Cd toxicity stress, the essentiality of this PC function remains to be shown. For metal(loid)s other than As and Cd the affinity to PCs and/or the induction of PC synthesis is too low to be relevant under natural conditions (e.g., Maitani et al., 1996; Satofuka et al., 2001; Schat et al., 2002). There is also a report that PCs would be involved in Zn detoxification (Tennstedt et al., 2009). But in this study, Zn hypersensitivity upon PC synthase knockout was observed only in yeast cells where the main Zn detoxification mechanism (Zn homeostasis factor, Zhf, Clemens et al., 2002) had been knocked out as well, and in *Arabidopsis thaliana* plants grown with 50 μM Zn, i.e., concentrations above the range this species may encounter in nature.

Interestingly, PCs are not required for metal tolerance in hypertolerant plants. In such plants (regardless of whether they are hyperaccumulators or not), the inhibition of PC synthase did not change the tolerance toward metals significantly (Schat et al., 2002). Comparing different ecotypes of *N. caerulescens*, these authors furthermore showed that in the non-Cd-hyperaccumulating, Cd-sensitive populations blockage of PC synthesis made the already Cd-sensitive populations (and only those) even more sensitive to Cd toxicity, i.e., they behaved like all non-hypertolerant/non-accumulator other species would do. Additionally, metal hyperaccumulator plants were found to have lower PC levels than related non-accumulator plants (Ebbs et al., 2002). Furthermore, in contrast to MTs (see above), PCs do not seem to be involved in binding of the non-hyperaccumulated but toxic copper in the Cd/Zn hyperaccumulator *N. caerulescens* (Mijovilovich et al., 2009). This could again be shown by EXAFS spectroscopy. Copper PCs have a completely different EXAFS spectrum than Cu-MTs, with only one strong Cu–Cu interaction peak at 2.56 Å and a weak contribution at 4.04 Å (Polette et al., 2000), but no contribution at distances where peaks in the Fourier transforms were observed (Mijovilovich et al., 2009).

### GLUTATHIONE

Glutathione is the main building block which PC synthase uses for making PCs. Besides this indirect involvement in metal detoxification, there are also some indications that glutathione itself acts as a low-affinity (monodentate) ligand for Cd. Pietrini et al. (2003) have found, using 50–100 μM cadmium to induce toxicity in *Phragmites australis*, a 30-fold increase in the concentration of reduced glutathione in leaves. In contrast, cadmium and copper at more realistic concentrations (0.03–3 μM total dissolved Cd) that already caused a strong induction of PCs in phytoplankton did not lead to upregulation of glutathione synthesis under metal stress conditions (Ahner et al., 2002). In hyperaccumulators, no upregulation of glutathione levels in response to metal uptake has been reported so far, and (as mentioned before) thiol ligands are generally not much involved in metal storage in hyperaccumulators (As: Wang et al., 2002; Cd: Küpper et al., 2004; Cu: Küpper et al., 2009; Ni: Sagner et al., 1998; Zn: Salt et al., 1999). However, a constitutive high level of glutathione biosynthesis may contribute to protection against reactive oxygen species (not metal complexation) in Ni hyperaccumulators belonging to the *Noccaea* (formerly *Thlaspi*) genus (Freeman et al., 2004).

### OTHER LIGANDS

As an exception to the rule of storage of hyperaccumulated elements predominantly in association with organic acids (see above), in selenium hyperaccumulators Se was found to be mainly stored as methylselenocysteine and g-glutamyl-methylselenocysteine (Freeman et al., 2006).

## COMPARTMENTATION

### ENHANCED TRANSLOCATION OF METALS FROM ROOTS TO THE SHOOT

While metal uptake through the root is the first important step in hyperaccumulation in land plants, most of the metal is stored in the shoots. Storage in the above-ground tissues is part of the definition of hyperaccumulators, and since the original discovery almost 150 years ago (Sachs, 1865) many studies throughout many decades have shown this phenotype (reviews, e.g., by Baker, 1981; Brooks, 1998; Küpper and Kroneck, 2005). As a particularly detailed recent long-term study dealing with cadmium uptake, Lovy et al. (2013) show that in the Cd/Zn hyperaccumulator *N. caerulescens*, exposed to constant Cd concentrations throughout the complete growth cycle, 86% of the Cd taken up was allocated to the shoots.

As a prerequisite for efficient transport from the roots to the above-ground parts of the plant, in numerous studies an enhanced uptake of metals into the root symplasm was found. In the Cd/Zn hyperaccumulator *N. caerulescens* compared to the related non-accumulator, *N. arvense* (Lasat et al., 1996, 1998), the hyperaccumulator showed a much higher translocation efficiency and further, a reduced sequestration into the root vacuoles was associated with the higher root to shoot translocation efficiency of *N. caerulescens* (Shen et al., 1997; Lasat et al., 1998; Zhao et al., 2006). Also in the *S. alfredii *(Crassulaceae)*, *found on highly Cd/Zn contaminated soils in the Zhejiang Province of China, Cd/Zn-hyperaccumulating and non-accumulating populations have been compared. This showed a very efficient xylem loading of zinc, leading to sequestration to the shoot in the hyperaccumulating populations (Lu et al., 2013), while in the non-accumulating populations more of the metal was found in the roots.

The early finding that root uptake and root to shoot translocation are strongly elevated in hyperaccumulators compared to non-accumulators (see above) got a genetic basis in recent years, as it was found that the heavy metal ATPase HMA4 is strongly overexpressed in roots of the Cd/Zn hyperaccumulator plants *Arabidopsis halleri* and *N. caerulescens* (Bernard et al., 2004; Papoyan and Kochian, 2004; Weber et al., 2004), and that this is linked to HMA4 gene multiplication (Talke et al., 2006; Hanikenne et al., 2008; O’Lochlainn et al., 2011). Overexpression experiments strongly suggest that HMA4 functions in loading Cd and Zn into the xylem (Verret et al., 2004; Hanikenne et al., 2008). This conclusion is further supported by the finding that expressing it in yeast causes efflux (Papoyan and Kochian, 2004), because loading of the dead xylem tubes always means efflux from the helper cells surrounding them, where HMA4 is most highly expressed (Hanikenne et al., 2008). Also in rice (*Oryza sativa)* HMAs play an important role in the transport of Cd and Zn (for a review, see Takahashi et al., 2012), making these proteins an interesting topic to study, as in contrast to hyperaccumulators, rice is one of the most important cereals worldwide. An exception from the rule of active enhanced root to shoot transport in hyperaccumulators seems to be Al, which is most likely transported apoplastically (Matsumoto et al., 1976).

### TRANSPORT TOWARD THE FINAL STORAGE CELLS VIA OVEREXPRESSED TRANSPORT PROTEINS

Xylem loading and xylem transport and thus distribution of metals away from the roots and toward the final storage sites are key steps in metal hyperaccumulation, as it was commented by White et al. (2002).

It has been found that metal hyperaccumulation is mediated, at least in part, by an up to 200 times higher expression of various metal transporter genes in hyperaccumulators compared to related non-accumulator plants (Pence et al., 2000; Assunção, 2001; Becher et al., 2004; Papoyan and Kochian, 2004; Hanikenne et al., 2008, reviewed by Verbruggen et al., 2009). As the name suggests, hyperaccumulators are characterized by strong accumulation, compared to the substrate, of a certain element. This is usually expressed as the bioaccumulation factor, which is commonly defined as the ratio of the concentration of an element inside the organism compared to the metal concentration in the substrate. To achieve a bioaccumulation coefficient far greater than one [characterizing hyperaccumulators as revealed already by their first discovery almost 150 years ago (Sachs, 1865)], the metals have to be pumped into their storage sites against the concentration gradient. Altogether, most likely many steps of the metal transport from root uptake until passage over the vacuolar membrane of the storage cells are against the concentration gradient. Therefore, all these transport steps require an active, i.e., energy consuming transport system (Salt and Wagner, 1993). Furthermore, specificity of the transport is tightly controlled. This is obvious in *N. caerulescens*, which hyperaccumulates Cd and Zn but does not accumulate copper when it is supplied to the nutrient solution at the same concentration (Mijovilovich et al., 2009). Furthermore, this species is, apart from some more resistant individuals, highly sensitive toward copper (Mijovilovich et al., 2009). This copper sensitivity is a severe problem for phytoremediation of sites that are contaminated with a combination of several metals (Walker and Bernal, 2004)

As just discussed, during the last decade numerous studies have been undertaken successfully to identify genes that are involved in metal transport in hyperaccumulators. Attempts to isolate and characterize the corresponding proteins where less successful, due to the often cysteine-rich sequences of metal-binding and metal transport proteins. Because of this high cysteine content making them prone to oxidation, these proteins tend to be extremely unstable once taken out of their natural membrane environment, rendering them difficult to handle during characterization studies (Parameswaran et al., 2007; Leitenmaier et al., 2011). Nevertheless, also on the protein level there are now a few studies available, yielding insight into function and properties of such proteins and showing how also other metal transport proteins, which are highly expressed in hyperaccumulators, should be characterized in the future. Investigations of the biochemical and biophysical properties of the *N. caerulescens* version of HMA4, NcHMA4, have shown that the ATPase function of this transporter is activated most strongly by Cd and Zn. Gels and western blots (using an antibody specific for NcHMA4) of crude root extract and of the purified protein revealed a size of NcHMA4 of about 50–60 kDa, while the mRNA for the NcHMA4 gene predicts a single protein with a size of 128 kDa. This indicates the occurrence of post-translational processing (Parameswaran et al., 2007). Whether this shortened but functional protein is the product of the C-terminally truncated copies of the gene (Craciun et al., 2012) still remains unknown. In recent work by Leitenmaier et al. (2011), NcHMA4 showed activity of NcHMA4 with Cu^2^^+^, Zn^2^^+^, and Cd^2^^+^ under various concentrations (0.03–10 μM tested), and all three metal ions activated the ATPase at a concentration of 0.3 μM, while there were clear differences in activation energy (E_A_) observed, depending on the metal applied. According to EXAFS, NcHMA4 appeared to bind Cd mainly by thiolate sulfur from cysteine, and not by imidazole nitrogen from histidine. These properties correspond well with what is known from a study of a human copper ATPase, ATP7A (Hung et al., 2007). Maximal activation of NcHMA4 and HsATP7b occurred at about the same metal concentration, and also the measured turnover rates were similar (Leitenmaier et al., 2011 vs. Hung et al., 2007). Therefore, also the other properties, investigated in only one of these two existing biochemical studies of CPx-type ATPase holoproteins, should be at least somewhat similar in the other. In this way, research on plant metal transport proteins has also medical relevance as mutations of the human copper ATPases cause well-known deadly diseases (Menkes and Wilson disease).

The expression of HMA4 not only in the roots but also in shoots clearly shows that it must have more functions than xylem loading. Expression is similarly high also in shoots, again with a maximum in the vascular bundle, which could mean that this protein is involved in xylem unloading as well (Craciun et al., 2012). Such a role dual role both in loading and unloading of the xylem by the same protein could be achieved simply by insertion of the protein in either the xylem-facing side of these cells (→loading) or the opposite side (→unloading). How the passage through the mesophyll to the epidermal storage cells is accomplished is still an open and important question, as shown also by the fact that HMA4 overexpression in a non-accumulator causes this plant to poison itself due to lack of mechanisms detoxifying the Cd and Zn arriving in the shoots (Hanikenne et al., 2008). Uptake over the cytoplasmic membrane of the epidermal storage cells is not well-characterized either, but a recent study on the cellular distribution and regulation of gene expression levels (Küpper and Kochian, 2010) indicates genes that may be candidates for causing this difference in metal accumulation between epidermal cells: NcZNT5 was highly expressed in epidermal storage cells, much higher than in other cell types. The ZIP-family of transporters, to which ZNT5 belongs, is generally localized in the plasma membrane (review by Guerinot, 2000).

### FINAL STORAGE SITE: VACUOLES OF LARGE EPIDERMAL CELLS

Hyperaccumulators have to store the enormous amounts of taken up metal in a way that it does not harm important enzymes and especially not photosynthesis, therefore it is crucial to keep the metal concentration in the cytoplasm of mesophyll cells as low as possible. It makes sense for hyperaccumulating plants to store metal in the vacuoles because in this organelle only enzymes like phosphatases, lipases, and proteinases (Wink, 1993; Carter et al., 2004) are present, which have not been found to be a target of heavy metal toxicity. Additionally it has been shown that high amounts of metals are stored specifically in the vacuoles of large epidermal cells (Küpper et al., 1999, 2001; Frey et al., 2000), where no chloroplasts are located and therefore also during transport through the cytoplasm harboring the chloroplasts, photosynthesis cannot be inhibited. Preferential storage of hyperaccumulated metals in the epidermis has been shown for the majority of hyperaccumulator species and for elements as chemically diverse as Al, As, Cd, Ni, Se, and Zn (e.g., Küpper et al., 1999, 2001; Frey et al., 2000; Lombi et al., 2002; Carr et al., 2003; Bhatia et al., 2004; Bidwell et al., 2004; Broadhurst et al., 2004; Cosio et al., 2005; Freeman et al., 2006). The approximate volume of this storage site multiplied by the metal concentration in it (data, e.g., for Zn from Küpper et al., 2009) indicates that about 70% of the total accumulated zinc in mature leaves is stored in the epidermis of *N. caerulescens*, and it is likely similar for most other species investigated by the other authors listed above. If the storage capacity of the epidermis is exceeded, enhanced storage seems to occur in the mesophyll (Küpper et al., 2001). This leads to inhibition of photosynthesis especially in those cells that accumulate most of the toxic metal (Küpper et al., 2007).

In the vacuoles of the epidermal metal storage cells, heavy metal concentrations of several hundred millimoles per liter can be reached (Küpper et al., 2001, Küpper et al., 2009), which already indicated that hyperaccumulation must involve active pumping of the metals into specific storage sites. The alternative hypothesis that the metal enrichment in the epidermis is caused by the transpiration stream (i.e., influx of metal-containing water into the epidermis, and evaporation of the water) could be excluded by a study on epidermal protoplasts. The drastically different cadmium uptake rates into epidermal storage cells compared to mesophyll cells in this study clearly showed that the epidermal accumulation is due to differences in active metal transport and not differences in passive mechanisms like transpiration stream transport or cell wall adhesion (Leitenmaier and Küpper, 2011).

Furthermore, it turned out that the transport over the cytoplasmic membrane is faster than the sequestration into the vacuole, making the latter the rate-limiting step in epidermal accumulation. Altogether, it seems likely that the transport steps over the plasma and tonoplast membranes of leaf epidermal storage cells are driving forces behind the hyperaccumulation phenotype (Leitenmaier and Küpper, 2011). Protein families involved in vacuolar sequestration may be the Nramp’s (natural resistance-associated macrophage proteins), CDF’s (cation diffusion facilitator), and CAX’s (cation exchanger; reviewed by Hall and Williams, 2003) as well as CPx-type ATPases. Until now, already several transporters for vacuolar sequestration of zinc (and possibly cadmium) and nickel have been investigated and could be partially characterized (Van der Zaal et al., 1999; Desbrosses-Fonrouge et al., 2005; Elbaz et al., 2006; Haydon and Cobbett, 2007; Morel et al., 2009). Several CDF transporters for vacuolar sequestration of Zn (and possibly Cd and Co) have been characterized, all are homologous, almost identical in sequence. These are MTP1, ZAT, and ZTP (Van der Zaal et al., 1999; Assunção, 2001; Becher et al., 2004; Dräger et al., 2004). The *Arabidopsis thaliana* homolog of this protein, AtMTP1, has been shown to mediate Zn detoxification and leaf Zn accumulation and it is known to be localized in the vacuolar (tonoplast) membrane (Desbrosses-Fonrouge et al., 2005). Due to the chemical similarity of cadmium and zinc, transporters designed for Zn inevitably will transport Cd as well for purely chemical reasons. MHX, a homolog of an *Arabidopsis thaliana* vacuolar metal (Fe, Mg, Zn) versus proton exchanger and member of the CDF protein family, was found to be highly expressed in the leaf vacuolar membrane of *Arabidopsis halleri* (Elbaz et al., 2006).

HMA3, a CPX- (=P_1B_)-type heavy metal ATPase, was found to mediate leaf vacuolar storage of Cd, Co, Pb, and Zn in *Arabidopsis thaliana* (Morel et al., 2009). The strongly elevated expression of HMA3 was shown to play a decisive role in Cd accumulation not only in *N. caerulescens* (Ueno et al., 2011b), but also in rice (Ueno et al., 2010, 2011a), reconfirming also the importance of the sequestration into vacuoles for the hyperaccumulation phenotype. However, the reported effects of the vacuolar sequestration on shoot metal concentrations as mediated by HMA3 are opposite in rice compared to *N. caerulescens*, because the localization of this expression is different. In rice, HMA3 sequesters Cd into root vacuoles, diminishing the Cd available for transport into the shoots (Ueno et al., 2010). In *N. caerulescens*, in contrast, it was shown to be expressed highly expressed also in the shoots, thus sequestering Cd there (Ueno et al., 2011b). For purely biophysical reasons (transport equilibria), this will cause enhanced xylem unloading in the shoots, and thus enhanced root–shoot translocation (i.e., a sink-driven transport).

The natural overexpression of NRAMPs was identified both in rice and in *N. caerulescens* to play an important role in Cd tolerance and possibly Cd accumulation (Oomen et al., 2009; Wei et al., 2009 and Takahashi et al., 2011).

Also at this final step, Al represents an exception: in the best known Al hyperaccumulator, *Camellia sinensis* (tea), Al is accumulated mostly in the cell walls, with very low concentrations inside the cells even when the samples are properly prepared to prevent artefactual cell wall localization (Carr et al., 2003).

### EXCEPTION: STORAGE IN THE MESOPHYLL

Although the final storage of metals in large epidermal cells is a typical phenomenon in most hyperaccumulators and for most hyperaccumulated metals, there are also exceptions. In the Zn hyperaccumulator *Arabidopsis halleri*, accumulating cadmium to a limited extent as well, except for a few trichomes epidermal cells are rather small. Therefore, despite their high concentrations with almost all the Zn and Cd being accumulated in a narrow ring in the trichome base, epidermal cells contribute only a minor proportion to total storage of Cd and Zn in this species while most of the Cd is stored in the photosynthetic mesophyll (Küpper et al., 2000). Although *Arabidopsis halleri* still accumulates Cd in the hyperaccumulation range, this may be the reason why it suffers from toxicity at much lower Cd concentrations (in the nutrient solution and inside the plant) than the strongest known Cd hyperaccumulator, *N. caerulescens, *ultimately limiting Cd accumulation in *Arabidopsis halleri*: Cd accumulation in the mesophyll represents a danger for Cd-induced inhibition of photosynthesis (Küpper et al., 2007). A seemingly similar situation was recently reported for the Cd/Zn hyperaccumulator *S. alfredii* (Yang et al., 2004), where Cd is accumulated in the mesophyll of leaves beside the pith and cortex of stems (Tian et al., 2011). Comparing this to other hyperaccumulators, however, it has to be kept in mind that *S. alfredii* as a Crassulaceae, has rather thick, succulent leaves, which means it has exceptionally large vacuoles in the mesophyll, making Cd storage there safer than it would be in regular sized mesophyll cells as they occur in *N. caerulescens* or *Arabidopsis halleri*. Another exception in the sense of hyperaccumulation in the mesophyll instead of the epidermis is manganese. In Mn-hyperaccumulating (*Gossia bidwillii*, Myrtaceae; *Virotia neurophylla*, Proteaceae) as well as strongly accumulating species (*Macadamia integrifolia*, *M. tetraphylla* (Proteaceae), it has been shown not to be translocated into the epidermis but to be primarily sequestrated into multiple palisade cell layers (the mesophyll), which contain photosynthetically active cells (Fernando et al., 2006a,b). However, in one accumulating species, *Grevillea exul* (Proteaceae), Fernando et al. (2008) showed a higher manganese content in the epidermal tissues, compared to the photosynthetic active tissues. The latter authors furthermore discussed that the translocation into photosynthetic active tissues might be due to their high need for manganese as part of the active center of the oxygen-evolving-complex (OEC). The sequestration into vacuoles, no matter if they are located in a storage cell or not and also independent of the type of metal, is always a transport against the concentration gradient and therefore needs an active transport system (Salt and Wagner, 1993, for a recent review see Martinoia et al., 2012).

## Conflict of Interest Statement

The authors declare that the research was conducted in the absence of any commercial or financial relationships that could be construed as a potential conflict of interest.

## Abbreviations

CAX: cation exchanger
CDF: cation diffusion facilitator
CPx-type: ATPases adenosine-tris-phosphatases with the specific intramembranous amino acid motif cysteine– proline– cysteine or cysteine– proline– histidine
E_ A_: energy of activation in kJ.mol-1
EDTA: ethylenediaminetetraacetic acid
EXAFS: extended X-ray absorption fine structure
HMA: heavy metal ATPase
mRNA: messenger RNA (Ribonucleic acid)
Nramps: natural resistance-associated macrophage proteins
XAS: X-ray absorption spectroscopy
